# CD34 and CD38 are prognostic biomarkers for acute B lymphoblastic leukemia

**DOI:** 10.1186/s40364-016-0080-5

**Published:** 2016-12-16

**Authors:** Zhiwu Jiang, Di Wu, Shouheng Lin, Peng Li

**Affiliations:** 1 State Key Laboratory of Respiratory Disease, Guangzhou Institutes of Biomedicine and Health, Chinese Academy of Sciences, 190 Kaiyuan Avenue, Science Park, Guangzhou, Guangdong 510530 China; 2 Key Laboratory of Regenerative Biology, South China Institute for Stem Cell Biology and Regenerative Medicine, Guangzhou Institutes of Biomedicine and Health, Chinese Academy of Sciences, Guangzhou, 510530 China; 3 Guangdong Provincial Key Laboratory of Stem Cell and Regenerative Medicine, South China Institute for Stem Cell Biology and Regenerative Medicine, Guangzhou Institutes of Biomedicine and Health, Chinese Academy of Sciences, Guangzhou, 510530 China

## Abstract

CD34 and CD38 proteins are usually used as surface markers to identify HSCs and Leukemic stem cells. However, there have been cases that lacked CD34 or CD38 protein but still had leukemia initiating capacity in B-ALL suggesting the restrictive of these two markers. CD34 and CD38 expression were detected in most B-ALL and can serve as a specific biomarker for the prognosis of this subset of leukemia. Lack of CD34 or high CD38 expression is associated with favorable prognosis.

## Background

Acute lymphocytic leukemia (ALL) is the most common hematological malignancy in childhood and accounts for about 20% of acute leukemia in adults [1–3]. On the basis of ontogenic classification, ALL is divided into T-lineage ALL and B-lineage ALL. B-lineage ALL is characterized by the expression of the B-cell markers CD19, CD22 and CD79a [4]. These cells lack expression of CD3 and of myeloperoxidase. Leukemic cells express various markers of differentiation that allow the distinction of at least three main subtypes: Early precursor B-ALL (pro-B-ALL, CD10^−^, CD19^+^, cCD79a^+^, cCD22^+^, TdT^+^), Common B-ALL (CD10^+^) and Precursor B-ALL (pre-B-ALL, cytoplasmic μ^+^, sIg^−^, CD10^+^/^−^) [5]. Clonal rearrangement analysis of immunoglobulin genes and TCR has demonstrated the single-cell origin of ALL. Leukemogenesis is a multistep process that requires the accumulation of alterations in a hematopoietic progenitor cell at multiple stages. The leukemic stem cell (LSC) hypothesis postulates that leukemia are hierarchically organized and leukemic stem cells have the capacity to self-renew, give rise to more differentiated progeny and maintain the leukemia long-term [6–8]. CD34^+^CD38^−^ cells have been shown to be able to initiate acute myeloid leukemia (AML) [9]. Similarly, previous studies have reported that in B-ALL, only CD34^+^CD38^−^ cells can initiate leukemia in immunodeficient recipients [10]. Recently, studies have revealed that the frequency of leukemia-initiating cells (LICs) is high and consistent between different stages of immunophenotypic maturation in B-ALL [11, 12]. Therefore, CD34 or CD38 may serve as potential biomarkers of LSCs in B-ALL cells.

### CD34 and CD38

CD34 is a cluster-of-differentiation molecule first described by Civin et al. in a cell surface glycoprotein [13] and is encoded by the *CD34* gene [14]. Two transcript variants encoding different isoforms have been found for this gene. The isoform a (b) gene has 2612 (2816) bases, localized on chromosome 1q32. CD34 isoform a (b) protein has 385 (328) amino acid residues. The CD34 protein plays a role in the attachment of stem cells to the bone marrow extracellular matrix or to stromal cells [15, 16].

CD34 is a transmembrane protein that was first identified on hematopoietic stem and progenitor cells. CD34 is one of the most widely used markers of hematopoietic stem cells (HSCs) and is involved in the inhibition of HSC differentiation, HSC expansion, signaling transduction and anti-adhesion [15].

CD38 is a type II glycoprotein that was originally described as a lymphoid cell surface differentiation marker [17]. The CD38 gene has 5694 bases, maps to chromosome 4p15 and shares a long evolutionary history with CD157. The CD38 protein has 300 amino acid residues. CD38 is also a multifunctional ectoenzyme that catalyzes the synthesis and hydrolysis of cyclic ADP-ribose (cADPR) from NAD^+^ to ADP-ribose. These reaction products are essential for the regulation of intracellular Ca^2+^. CD38 has been used as a prognostic marker in leukemia [18].

### CD34 and CD38 as biomarkers for LICs

The CD34^+^/CD38^−^ immunophenotype is used to identify HSCs and LICs in AML [19, 20]. There is an ongoing debate over the existence of LICs in human B-ALL. In 2000, Cobaleda et al. transplanted Philadelphia chromosome-positive (Ph^+^) ALL cells into non-obese diabetic/severe combined (NOD/SCID) mice and found that only the CD34^+^CD38^−^ fraction could give rise to ALL [10]; the CD34^+^CD38^+^ and CD34^−^ fractions contained no cells with this capability. By adding cytokines, Blair and colleagues successfully educated B-ALL cells in serum-free cell culture. They fractionated the B-ALL cells on the basis of cell-surface-marker expression and demonstrated that the ALL cells with long-term proliferative and replating potential were CD34^+^CD10^−^CD19^−^ [21]. Using the newborn NOD/SCID/IL2Rg (null) xenotransplantation model, Ishikawa et al. demonstrated that CD38 expression was irrelevant in identifying a leukemogenic population in human primary B-ALL. By intravenously injecting purified CD34^+^CD38^+^CD19^+^ or CD34^+^CD38^−^CD19^+^ cells from B-precursor ALL patients, they successfully demonstrated the leukemia-initiating capacity of both cell types [22]. All of these studies reached the unanimous verdict that it was the expression of CD34 that highlighted the LIC population, similar to the case of AML. However, Vormoor et al. and our studies established that blast cells from different stages of acute lymphoblastic leukemia were all able to reconstitute the complete leukemia phenotype in vivo, no matter in which subpopulations they were involved (CD34, CD38, CD20 or CD19, either positive or negative) [11, 23]. These conflicting results indicate that key questions regarding the markers of LSCs in ALL remain unresolved.

### CD34 and CD38 as biomarkers for prognosis in B-ALL

CD34^+^CD38^−^ B-ALL cells have been used not only in studies of LSCs, but also in diagnosis and prognosis (Table 1). In a large cohort of 2028 children with ALL, the treatment outcomes of a subset of B-lineage ALL patients with CD10^+^CD19^+^CD34^+^ immature B-progenitor leukemia were compared with the treatment outcomes of the remaining CD19^+^ B-lineage ALL patients. It was found that the CD10^+^CD19^+^CD34^+^ immature B-progenitor patients were associated with a more favorable prognosis [24]. In a recent study of 112 cases of childhood ALL, high proportions of CD34^+^CD38^−^ cells were positively correlated with high-risk subgroups and negatively correlated with outcomes [25]. A study of 63 cases of ALL found that CD34 protein expression was higher in pediatric B-ALL than in T-ALL [26]. In another study, 75 newly diagnosed adult ALL patients were analyzed for CD34 expression. Expression of this protein was positively associated with B-cell-lineage ALL. CD34 was more expressed in poor-risk B-ALL patients and associated with features of poor prognosis [27]. Hence, the CD34 expression pattern may have a prognostic utility in B-ALL.

**Table 1:** Summary of the CD34 or CD38 related cell subsets corresponding with B-ALL prognosis

Cell Subset	Prognosis	Reference
CD34 +	positive/negative	CD34 expression is favorable and significant prognosis in childhood B-lineage ALL (age 1 to 10 years). [26] CD34 mostly expressed in poor risk adult B-ALLs patients, characterized with higher WBC cell count, and higher percentage of peripheral blood leukemic cells [27]
CD38 +	positive	Ph + ALL showed lower CD38 expression than Ph- groups. Overall survival favored ALL patients with higher CD38 levels [26]
CD34 + CD38− cells	negative	A high proportion of CD34+/CD38- cells is associated with clinical and biological features and a poor prognosis of childhood ALL. [25]
CD10 + CD19 + CD34+	positive	With CD10 + CD19’CD34+ immature B-progenitor subset, pediatric B-lineage ALL patients may have favorable characteristics to achieve favorable EFS outcomes. [24]

In contrast to CD34 expression, increased CD38 expression in acute leukemia (AML or ALL) is associated with favorable prognosis [18]. In a study of 304 AML and 138 ALL patients, Ph^+^ ALL patients showed lower CD38 expression compared with other cytogenetic groups [28]. CD38 expression was found to be positively related to overall survival rates among AML and ALL patients. Therefore, these studies suggest that higher expression of CD34 and CD38 may represent prognostic biomarkers of poor and favorable outcomes for B-ALL, respectively.

## Conclusion

The CD34 and CD38 patterns in B-ALL cannot be used as specific surface markers to distinguish LSCs from blast cells. CD34 and CD38 expression are detected in most cases of B-ALL and can serve as specific biomarkers for prognosis. Lack of CD34 or high CD38 expression is associated with favorable prognosis.

## Abbreviations

ALL: Acute lymphoblastic leukemia
AML: Acute myeloid leukemia
LICs: Leukemia initiating cells
LSCs: Leukemia stem cells

## References

[1] Liew E, Atenafu EG, Schimmer AD, Yee KW, Schuh AC, Minden MD, Gupta V, Brandwein JM (2012). Outcomes of adult patients with relapsed acute lymphoblastic leukemia following frontline treatment with a pediatric regimen. Leuk Res.

[2] Larson S, Stock W (2008). Progress in the treatment of adults with acute lymphoblastic leukemia. Curr Opin Hematol.

[3] Pui CH, Evans WE (2006). Treatment of acute lymphoblastic leukemia. N Engl J Med.

[4] Pui CH, Robison LL, Look AT (2008). Acute lymphoblastic leukaemia. Lancet.

[5] NCCN Clinical Practice Guidlines in Oncology-Acute Lymphoblastic Leukemia (2016 Version I) [DB/OL]. https://www.nccn.org. Accessed 6 Dec 2016.

[6] Magee JA, Piskounova E, Morrison SJ (2012). Cancer stem cells: impact, heterogeneity, and uncertainty. Cancer Cell.

[7] Clarke MF, Dick JE, Dirks PB, Eaves CJ, Jamieson CHM, Jones DL, Visvader J, Weissman IL, Wahl GM (2006). Cancer Stem Cells—Perspectives on Current Status and Future Directions: AACR Workshop on Cancer Stem Cells. Cancer Res.

[8] Huntly BJ, Gilliland DG (2005). Leukaemia stem cells and the evolution of cancer-stem-cell research. Nat Rev Cancer.

[9] Bonnet D, Dick JE (1997). Human acute myeloid leukemia is organized as a hierarchy that originates from a primitive hematopoietic cell. Nat Med.

[10] Cobaleda C, Gutierrez-Cianca N, Perez-Losada J, Flores T, Garcıa-Sanz R, Gonzalez M, Sánchez-Garcıa I (2000). A primitive hematopoietic cell is the target for the leukemic transformation in human philadelphia-positive acute lymphoblastic leukemia. Blood.

[11] le Viseur C, Hotfilder M, Bomken S, Wilson K, Röttgers S, Schrauder A, Rosemann A, Irving J, Stam RW, Shultz LD (2008). In childhood acute lymphoblastic leukemia, blasts at different stages of immunophenotypic maturation have stem cell properties. Cancer Cell.

[12] Rehe K, Wilson K, Bomken S, Williamson D, Irving J, den Boer ML, Stanulla M, Schrappe M, Hall AG, Heidenreich O (2013). Acute B lymphoblastic leukaemia-propagating cells are present at high frequency in diverse lymphoblast populations. EMBO Mol Med.

[13] Civin CI, Strauss LC, Brovall C, Fackler MJ, Schwartz JF, Shaper JH (1984). Antigenic analysis of hematopoiesis. III. A hematopoietic progenitor cell surface antigen defined by a monoclonal antibody raised against KG-1a cells. J Immunol.

[14] Satterthwaite AB, Burn TC, Le Beau MM, Tenen DG (1992). Structure of the gene encoding CD34, a human hematopoietic stem cell antigen. Genomics.

[15] Furness SG, McNagny K (2006). Beyond mere markers: functions for CD34 family of sialomucins in hematopoiesis. Immunol Res.

[16] Nielsen JS, McNagny KM (2008). Novel functions of the CD34 family. J Cell Sci.

[17] Orciani M, Trubiani O, Guarnieri S, Ferrero E, Di Primio R (2008). CD38 is constitutively expressed in the nucleus of human hematopoietic cells. J Cell Biochem.

[18] Deaglio S, Mehta K, Malavasi F (2001). Human CD38: a (r) evolutionary story of enzymes and receptors. Leuk Res.

[19] Wang L, Gao L, Xu S, Gong S, Chen L, Lu S, Chen J, Qiu H, Xu X, Ni X (2013). FISH + CD34 + CD38- cells detected in newly diagnosed acute myeloid leukemia patients can predict the clinical outcome. J Hematol Oncol.

[20] Yan Y, Wieman EA, Guan X, Jakubowski AA, Steinherz PG, O’Reilly RJ (2009). Autonomous growth potential of leukemia blast cells is associated with poor prognosis in human acute leukemias. J Hematol Oncol.

[21] Cox CV, Evely RS, Oakhill A, Pamphilon DH, Goulden NJ, Blair A (2004). Characterization of acute lymphoblastic leukemia progenitor cells. Blood.

[22] Hong D, Gupta R, Ancliff P, Atzberger A, Brown J, Soneji S, Green J, Colman S, Piacibello W, Buckle V (2008). Initiating and cancer-propagating cells in TEL-AML1-associated childhood leukemia. Science.

[23] Jiang Z, Deng M, Wei X, Ye W, Xiao Y, Lin S, Wang S, Li B, Liu X, Zhang G (2016). Heterogeneity of CD34 and CD38 expression in acute B lymphoblastic leukemia cells is reversible and not hierarchically organized. J Hematol Oncol.

[24] Uckun FM, Sather H, Gaynon P, Arthur D, Nachman J, Sensel M, Steinherz P, Hutchinson R, Trigg M, Reaman G (1997). Prognostic significance of the CD10 + CD19 + CD34+ B-progenitor immunophenotype in children with acute lymphoblastic leukemia: a report from the Children’s Cancer Group. Leuk Lymphoma.

[25] Long J, Liu S, Li K, Zhou X, Zhang P, Zou L (2014). High proportion of CD34+/CD38-cells is positively correlated with poor prognosis in newly diagnosed childhood acute lymphoblastic leukemia. Leuk Lymphoma.

[26] Pui CH, Hancock ML, Head DR, Rivera GK, Look AT, Sandlund JT, Behm FG (1993). Clinical significance of CD34 expression in childhood acute lymphoblastic leukemia. Blood.

[27] Thomas X, Archimbaud E, Charrin C, Magaud JP, Fiere D (1995). CD34 expression is associated with major adverse prognostic factors in adult acute lymphoblastic leukemia. Leukemia.

[28] Keyhani A, Huh YO, Jendiroba D, Pagliaro L, Cortez J, Pierce S, Pearlman M, Estey E, Kantarjian H, Freireich EJ (2000). Increased CD38 expression is associated with favorable prognosis in adult acute leukemia. Leuk Res.

